# A comparison of passive and active dust sampling methods for measuring airborne methicillin-resistant *Staphylococcus aureus* in pig farms

**DOI:** 10.1093/annweh/wxad033

**Published:** 2023-06-10

**Authors:** Anne E Rittscher, Abel A Vlasblom, Birgitta Duim, Peter Scherpenisse, Isabella J van Schothorst, Inge M Wouters, Liese Van Gompel, Lidwien A M Smit

**Affiliations:** Institute for Risk Assessment Sciences (IRAS), Faculty of Veterinary Medicine, Utrecht University, Yalelaan 2, 3584 CM Utrecht, The Netherlands; Department of Infectious Diseases and Immunology (I&I), Faculty of Veterinary Medicine, Utrecht University, Yalelaan 1, 3584 CL, Utrecht, The Netherlands; Department of Infectious Diseases and Immunology (I&I), Faculty of Veterinary Medicine, Utrecht University, Yalelaan 1, 3584 CL, Utrecht, The Netherlands; Institute for Risk Assessment Sciences (IRAS), Faculty of Veterinary Medicine, Utrecht University, Yalelaan 2, 3584 CM Utrecht, The Netherlands; Institute for Risk Assessment Sciences (IRAS), Faculty of Veterinary Medicine, Utrecht University, Yalelaan 2, 3584 CM Utrecht, The Netherlands; Institute for Risk Assessment Sciences (IRAS), Faculty of Veterinary Medicine, Utrecht University, Yalelaan 2, 3584 CM Utrecht, The Netherlands; Institute for Risk Assessment Sciences (IRAS), Faculty of Veterinary Medicine, Utrecht University, Yalelaan 2, 3584 CM Utrecht, The Netherlands; Institute for Risk Assessment Sciences (IRAS), Faculty of Veterinary Medicine, Utrecht University, Yalelaan 2, 3584 CM Utrecht, The Netherlands

**Keywords:** airbourne dust, antimicrobial resistance, electrostatic dust collectors, MRSA, occupational exposure, pig farms, respiratory exposure

## Abstract

Methicillin-resistant strains of *Staphylococcus aureus* (MRSA) are resistant to most β-lactam antibiotics. Pigs are an important reservoir of livestock-associated MRSA (LA-MRSA), which is genetically distinct from both hospital and community-acquired MRSA. Occupational exposure to pigs on farms can lead to LA-MRSA carriage by workers. There is a growing body of research on MRSA found in the farm environment, the airborne route of transmission, and its implication on human health. This study aims to directly compare two sampling methods used to measure airborne MRSA in the farm environment; passive dust sampling with electrostatic dust fall collectors (EDCs), and active inhalable dust sampling using stationary air pumps with Gesamtstaubprobenahme (GSP) sampling heads containing Teflon filters. Paired dust samples using EDCs and GSP samplers, totaling 87 samples, were taken from 7 Dutch pig farms, in multiple compartments housing pigs of varying ages. Total nucleic acids of both types of dust samples were extracted and targets indicating MRSA (*femA, nuc, mecA*) and total bacterial count (16S rRNA) were quantified using quantitative real-time PCRs. MRSA could be measured from all GSP samples and in 94% of the EDCs, additionally MRSA was present on every farm sampled. There was a strong positive relationship between the paired MRSA levels found in EDCs and those measured on filters (Normalized by 16S rRNA; Pearson’s correlation coefficient *r* = 0.94, Not Normalized; Pearson’s correlation coefficient *r* = 0.84). This study suggests that EDCs can be used as an affordable and easily standardized method for quantifying airborne MRSA levels in the pig farm setting.

**“What’s important about this paper”**

This study found a significant positive association between the Methicillin-resistant *Staphylococcus aureus* (MRSA) levels in pig stables measured by active air sampling, using stationary air pumps with Gesamtstaubprobenahme (GSP) sampling heads, and those measured with Electrostatic Dust fall Collectors (EDCs). This demonstrates that EDCs are a feasible, affordable, and easily standardized way to measure MRSA in airborne farm dust. This finding is important as measuring MRSA is of great public health relevance, and EDCs could be a valuable tool in making large-scale surveillance feasible.

## Introduction

Methicillin-resistant strains of *Staphylococcus aureus* (MRSA) are resistant to most β-lactam antibiotics, an antibiotic class that includes Penicillin (Curello and Macdougall, 2014). MRSA is an opportunistic pathogen which makes it dangerous in healthcare settings; however, most people colonized with MRSA will not experience any health effects (Davis et al. 2004). Genetic studies have determined that the prevalence of MRSA infections in Europe has been increasing in people unconnected to hospitals (Gajdács 2019). This can be attributed in part to the spread of livestock-associated MRSA (LA-MRSA) strains (Kozaida et al. 2019; Sieber et al. 2019). LA-MRSA was first described in the Netherlands in 2004, and since then, pigs have been found to be an important LA-MRSA reservoir (Voss et al. 2005). People in close contact with pigs through living or working on farms can become colonized with MRSA, from which human-to-human spread can occur (Cuny et al. 2015). The concentration of airborne MRSA in farms is highly correlated to the MRSA loads found in the noses of exposed people (Angen et al. 2017). Other factors that influence MRSA transmission from animals to humans include the prevalence of MRSA in the animals, the number of hours spent in stables, and exposure to contaminated dust (Bos et al. 2016). In farms, airborne MRSA can be found as clusters of cells or attached to airborne particles such as fragments of skin cells or feed; this MRSA aggregates, then settles as dust, from which it can be cultured for up to 30 days (Feld et al. 2017; Madsen et al. 2018).

Previous studies seeking to analyze MRSA in dust have used a variety of methods making the available data difficult to interpret (Gilbert et al. 2012; Bos et al. 2016; Angen et al. 2017; Madsen et al. 2018; Kraemer et al. 2019; Sørensen et al. 2020). Of these studies, some have chosen to use active air sampling, which while valuable, can be expensive and labor intensive to gather (Gilbert et al. 2012; Angen et al. 2017; Madsen et al. 2018; Kraemer et al. 2019). Passive sampling using Electrostatic Dust fall Collectors (EDCs) could be a cost and labor-effective alternative and has been used by numerous studies in this setting in the past (Bos et al. 2016; Feld et al. 2017; Vestergaard et al. 2018; Madsen et al 2019). Previous studies have found EDCs to be useful and reproducible tools for measuring the microbial composition of dust found in farm environments (Normand et al. 2009; Bos et al. 2016). Additionally, EDCs are not size selective, they collect any size particle which is small enough to become airborne, potentially providing a more complete picture of the airborne microbial environment (Madsen et al. 2018; Viegas et al. 2019; de Rooij et al. 2021). The main aim of the study is to directly compare two common sampling methods to measure airborne MRSA in the farm environment: passive dust sampling with EDCs, and active inhalable dust sampling using GSPs. This information will help elucidate possible environmental reservoirs for MRSA in the pig farm environment and aid in the creation of accurate transmission risk models (Sørensen et al. 2020).

## Methods

Methods are briefly described here. More details can be found in the Supplementary Material.

### Study population

Pig farms were recruited from across the Netherlands. Farms could be of any size, and only farms using organic practices were excluded, as they were unlikely to be MRSA positive. A convenience sample of seven farms in total was applied. At each farm, an effort was made to conduct paired passive and active dust sampling in herds of diverse ages.

### Questionnaire data

Every farm owner was asked to fill out a 26-item general questionnaire about antimicrobial use and general biosecurity practices on the farm that could potentially influence the microbial diversity and MRSA abundance on the farm. The anonymized questionnaire data are available in the supplementary material, as well as being openly available in the Zenodo entry for this project.

### Passive dust sampling

EDCs contained two electrostatic cloths (polyester electrostatic cloth; Albert Heijn, Zaandam, The Netherlands) held by a plastic frame and were placed onto a cardboard platform and hung from the ceiling approximately 1.5 m from the ground (method adapted from Noss et al. 2008). An average of two EDCs frames (two cloths per frame) acted as duplicate measurements and were left in place for 7 days before being sent back by the farmer by mail to Utrecht University (Utrecht, the Netherlands) for laboratory analysis. A blank EDC was taken to each farm that remained sealed as a contamination control.

### Active air sampling

Active air sampling was performed using Gilian Gilair five pumps (3.5 l/min) along with Teflon filters (PE Drain Disc, Whatman: GE Healthcare, with Teflon 2.0 µm 37 mm filters: Pall Corporation) in Gesamtstaubprobenahme (GSP) sampling heads at a height of 1.5 m, hereafter referred to as GSP samplers. At each farm, a blank filter remained sealed and was used as a negative control. An average of active samplers was placed in each stable and acted as duplicates. Any peculiarities, such as malfunctioning pumps or torn filters, were recorded on the field forms. GSP samplers were left in place for 6 h and checked periodically.

### Laboratory methods

Once in the laboratory, both EDCs and Teflon filters were stored at −20°C, and thawed before DNA extraction, as described in Supplementary Material. Five microliters of DNA were used as template in quantitative real-time PCRs targeting *femA, nuc*, *mecA*, and 16S rRNA, with all samples being run in duplicate. All targets were detected using the LightCyler480 (Roche Molecular Biochemicals, Mannheim, Germany) and associated program.

### Statistical analysis

Statistical analysis was performed using RStudio V.4.2.2 (R Core Team 2022). The average Ct (Crossing Point) from the PCR replicates from the qPCR analysis was taken. Ct values over 40 were excluded, as they were considered outside of the reliable range of the PCR. PCR results are expressed in equivalent colony counts (CFUeq) calculated with a standard curve made with serial dilution of CFU counts from a reference MRSA strain (ST398). Total air volume sampled was calculated using flow rate and run time and used to calculate log_10_ MRSA CFUeq per cubic meter of air sampled for the active air samples. The log_10_ transformed MRSA CFUeq per square meter of surface area per day was used for EDCs. A mean MRSA count for each type of measurement was calculated from the duplicates taken in the same compartment.

As it is not possible to quantify MRSA presence with any one of the qPCR targets alone, MRSA abundance was estimated based on the presence of multiple targets. S. *aureus* count was estimated from the *nuc* or f*emA* count—whichever was highest—then the MRSA count was determined by taking the *S. aureus* count or *mecA* count whichever was lowest, as described in Bos et al. (2016).

In addition to absolute CFUeq, the relative abundance was estimated by dividing the gene counts by the sample’s corresponding 16S count (normalization). This method of estimating the relative abundance of genes was used by Luiken et al. (2022). Non-normalized results are a useful metric for inferring exposure risk, while results normalized for total bacterial counts help with comparing relative composition of dust collected using different sampling methods.

A Deming or orthogonal regression was performed on the paired data from active and passive air samples. A Deming regression analysis was used to account for error in both the *x* and *y* axis instead of the standard least squared regression that measures error only in the *y* axis.

## Results

### Farm characteristics

General farm characteristics are displayed in Table 1. On two farms only fattening pigs were present, while pigs of different ages were present on the other five farms.

**Table 1.** Overview of samples collected by active and passive air sampling in seven Dutch pig farms

Farm ID	Types of animals present	Average *N* of sows on the Farm per year	Average *N* of fatteners on the farm per year	EDCs (*N*)	Median MRSA as measured by EDC (log(CFUeq per m^2^ per day))	Stationary air samples (*N*)	Median MRSA as measured by GSP samplers (log(CFUeq per^3^ m))	Compartments sampled (*N*)
**1**	Piglets and Sows, Weaned Piglets, Fattening Pigs	127	40	6	2.90	2	3.11	3
**2**	Piglets and Sows, Weaned Piglets, Fattening Pigs	600	600	9	0.61	6	1.51	3
**3**	Piglets and Sows, Weaned Piglets, Fattening Pigs	440	3300	9	2.68	5^a^	3.42	3
**4**	Fattening Pigs	n/a	4960	6	2.01	4	2.47	2
**5**	Piglets and Sows, Weaned Piglets, Fattening Pigs	600	130	8	2.60	6	2.17	3
**6**	Piglets and Sows, Weaned Piglets, Sows	310	n/a	9	1.41	6	1.93	3
**7**	Fattening Pigs	n/a	11,400	6	2.11	4	2.86	2
**Total**	ND	ND	ND	53	ND	33	ND	19

^a^One stationary air sample value from Farm 3 was removed from data analysis due to a pump failure.

None of the farms reported antibiotic use in the week before sampling. No *S. aureus* (based on the *femA* and *nuc* count) was detected in any of the field blanks. MRSA could be quantified in all but 3 of the 87 EDCs and was found in all GSP samples and was present on every farm sampled.

### Correlation between PCR targets

We found a positive correlation between all three gene targets (*mecA, femA,* and *nuc*). The highest Pearson correlation coefficient was found between *femA* and *nuc* (*r* = 0.98, *P*-value <0.005) that indicates *S. aureus* presence in the sample. Lower correlations were found between *mecA* and *femA* (*r* = 0.35, *P*-value < 0.005), and *mecA* and *nuc* (*r* = 0.37 *P*-value <0.005) possibly indicating the presence of S*taphylococci* other than MRSA in the samples.

### Comparison of EDC and GSP sampler air measurements

**Figure 1.**
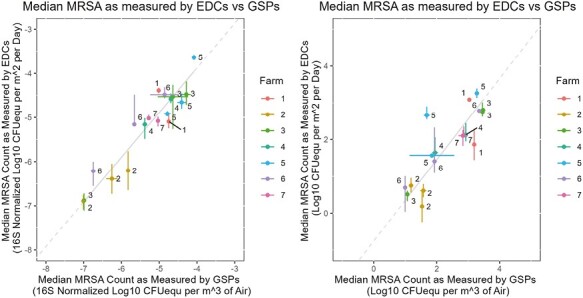
a) Median MRSA counts as measured by EDCs versus GSP samplers paired by compartment within each farm using the normalized data. The cross bars on each point indicate the maximum and minimum measurements taken in that compartment. The gray line indicates the Deming regression line. Normalized Line: Log10 EDC = 1.01 × Log10 GSP + 0.17. b) Median MRSA counts as measured by EDCs versus GSP samplers paired by compartment within each farm and non-normalized data. The cross bars on each point indicate the maximum and minimum measurements taken in that compartment. The gray line indicates the Deming regression line. Non-normalized Line: Log10 EDC = 1.10 × Log10 GSP − 0.76.

We found a positive relationship between relative MRSA loads measured in stationary air samples and the relative MRSA loads derived in the EDCs (Fig. 1a, Pearson *r* = 0.94, *P*-value <0.005; Deming orthogonal regression: β = 1.01 [CI: 0.85–1.18], *R*^2^ = 0.94). This result is also seen when a Deming orthogonal regression was done with the non-normalized data (Fig. 1b, Pearson *r* = 0.84, *P*-value <0.005; β = 1.10 [CI: 0.86–1.39], *R*^2^ = 0.84).

### Discussion and potential impact

This study demonstrates that EDCs are a feasible way to quantify MRSA in air samples from various pig farm environments and are a comparable alternative sampling method to active air sampling using GSP samplers. The high correlation coefficient between MRSA levels measured by the two methods as well as the linearity of the orthogonal regression model indicate that the relative ranking of results obtained by EDCs and GSP samplers are comparable.

#### Benefits of EDCs

One of the major advantages of EDCs is that they are affordable and easier to use on a large scale than active air sampling. EDCs can be used for prolonged periods of time to potentially reflect airborne levels in the setting on average, unlike active sampling which only captures a snapshot of the environment. This capacity for longer sampling times makes EDCs valuable for assessing the human health risks of occupational exposures. Additionally, EDCs are not size selective, which is particularly important for measuring MRSA in, as the cells can attach to airborne particles of various sizes (Madsen et al. 2018). Previous work has shown EDCs can also be used to measure other types of environmental exposures such as fungal contamination, bacterial endotoxin, allergens, and viral RNA (Cozen et al. 2008; Noss et al. 2008; Viegas et al. 2019; de Rooij et al. 2021). Frankel et al. (2012) compared multiple dust measurement methods to quantify airborne fungi and endotoxin. The study found that EDCs collected the most representative sample of airborne dust, and correlated most closely to GSP measurements when compared to the other passive samplers tested (Frankel et al. 2012). EDC’s capacity for measuring multiple types of exposures, including MRSA, could be utilized in future large-scale studies to get a complete picture of indoor airborne environments in occupational or residential settings.

While reproducibility was not directly measured in this study, EDCs have in the past been found to produce reproducible results during repeated sampling campaigns in the same setting (Noss et al. 2008; Normand et al. 2009). A future study that confirms this capacity for reproducibility of EDCs for MRSA measurements would be beneficial.

#### Potential impact

MRSA is a significant public health concern despite efforts to curb the rise of antimicrobial-resistant bacteria. In 2009, the Dutch government introduced a strict set of rules for antimicrobial use (AMU) in livestock, subsequently AMU in the industry fell by 59% between 2009 and 2014 (Speksnijder et al. 2015). Despite this, a study done in the Netherlands by Dierikx et al. (2016), found that all of the 56 batches of pigs tested at slaughterhouses were MRSA positive, therefore still posing a risk to the workers, in spite of the national decline in AMU.

Additionally, it is not yet known what “dose-response” effect reducing AMU in livestock will have on the presence of resistant bacteria in the air, this in conjunction with the knowledge that LA-MRSA has shown to be highly adaptable meaning that continual long-term monitoring will be important for years to come (Cuny et al. 2015; Speksnijder et al. 2015; Bosch et al. 2016). The use of cheap and easy-to-use airborne dust sampling tools such as EDCs will help facilitate this monitoring.

Our findings could aid in the development of models such as one created by Sørensen et al. in 2020 to study MRSA spread in pig farms and evaluate possible intervention strategies. The Sørensen model examines how different methods of reducing MRSA loads in stables limit the spread to humans and other farms, by modeling how MRSA spreads within a pig herd through contaminated air, which can be used as a proxy for human risk (Sørensen et al. 2020). Sørensen et al. (2020) note that one of the challenges in building the model was a lack of studies to compare the models’ predicted air concentrations of MRSA to. This is due in part to different methodologies being applied in the few studies available, making comparison difficult.

Our finding that the included passive and active air sampling methods result in strongly correlated estimates of MRSA concentration helps address this issue by making previous work done with these different methods more comparable and thus strengthening assumptions made in such risk models.

#### Limitations

One evident drawback of this study was the relatively small number of farms sampled. Despite its small size, this study sampled pigs at various ages and provides valuable information on the feasibility of large-scale EDC use, as well as finding a significant correlation between the sampling methods employed. Additionally, we only measured MRSA using molecular targets which can overestimate the amount of viable MRSA in the samples, because nonviable cells are also measured. This may have been alleviated in part by our use of a shorter sampling time, limiting cell die off, as well as by the formulas used to calculate CFUeq, which are based on standard curves made with viable cells (Madsen et al. 2019).

## Conclusion

Despite its small size, this study demonstrates that EDCs are an adequate sampling method for future large testing campaigns measuring airborne MRSA levels in pig farms. This study adds data to the existing body of work that indicates that EDCs are a feasible, and easily standardized way to measure airborne dust when compared to active air sampling using GSPs. Our data establishes that EDCs can be used accurately in a variety of pig stables with a range of airborne MRSA concentrations as a proxy for active air sampling.

## Supplementary Material

wxad033_suppl_Supplementary_Figure_S1

wxad033_suppl_Supplementary_Figure_S2

wxad033_suppl_Supplementary_Figure_S3

wxad033_suppl_Supplementary_Methods

wxad033_suppl_Supplementary_Questionnaire_Responses

## Data Availability

The data that supports the findings of this study are openly available in Zenodo at https://zenodo.org/record/7912764#.ZGMKRnZBw2w, 10.5281/zenodo.7912764
