# Supplementary figures and images for: A comparison of passive and active dust sampling methods for measuring airborne methicillin-resistant Staphylococcus aureus in pig farms
Source: Ann Work Expo Health. 2023 Jun 10;67(8):1004–10. doi: 10.1093/annweh/wxad033 (PMC10516621; doi:10.1093/annweh/wxad033)

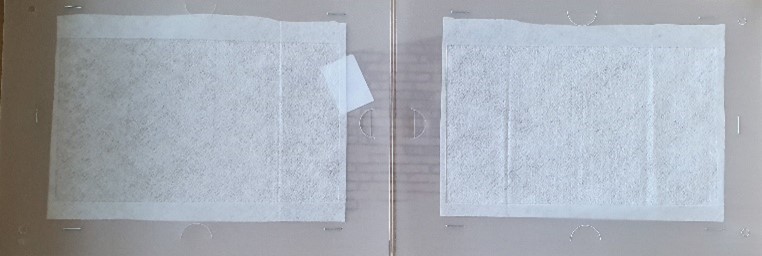

Supplement: wxad033_suppl_Supplementary_Figure_S1 [file wxad033_suppl_supplementary_figure_s1.jpeg]

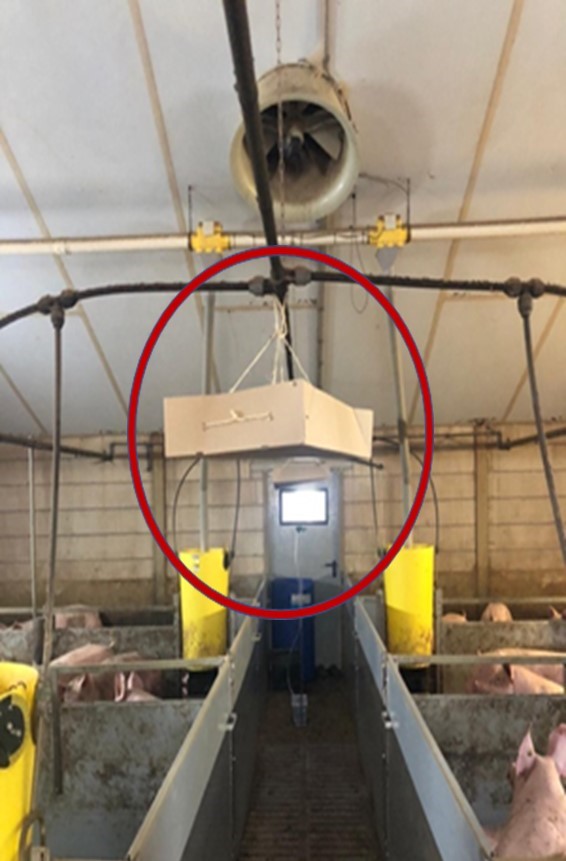

Supplement: wxad033_suppl_Supplementary_Figure_S2 [file wxad033_suppl_supplementary_figure_s2.jpeg]

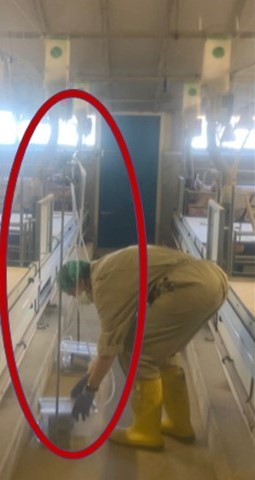

Supplement: wxad033_suppl_Supplementary_Figure_S3 [file wxad033_suppl_supplementary_figure_s3.jpeg]
