# Supplementary material for: A comparison of passive and active dust sampling methods for measuring airborne methicillin-resistant Staphylococcus aureus in pig farms
Source: Ann Work Expo Health. 2023 Jun 10;67(8):1004–10. doi: 10.1093/annweh/wxad033 (PMC10516621; doi:10.1093/annweh/wxad033)
Supplement: wxad033_suppl_Supplementary_Methods [file wxad033_suppl_supplementary_methods.docx]

# A Comparison of Passive and Active Dust Sampling Methods for Measuring Airborne MRSA in Pig Farms: Extended Method Supplement

*Authors: Anne Rittscher, Abel A. Vlasblom, Birgitta Duim, Peter Scherpenisse, Isabella van Schothorst, Inge M. Wouters, Liese Van Gompel, Lidwien A.M. Smit*

# Methods:

## Sampling and storage

### Electrostatic dust fall collectors (EDCs)


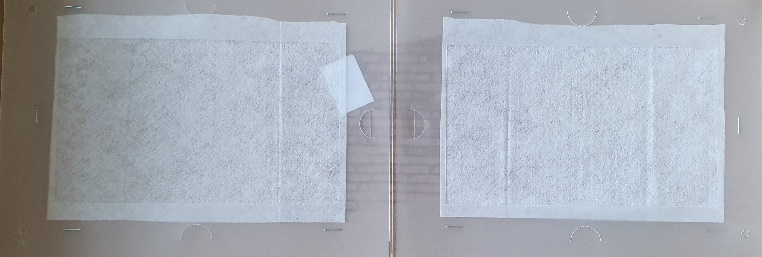
EDCs were used to sample settled dust in the pig farm stables. EDCs contained two sterilized electrostatic dust cloths (polyester electrostatic cloth) held by a plastic frame (Figure S1) and were placed onto a cardboard platform and hung from the ceiling (method adapted from Noss et al.,2008) Each side measured 0.2035m^2, resulting in a surface area of 0.407m^2 per sample.

Figure S1: An open EDC sample containing two cloths


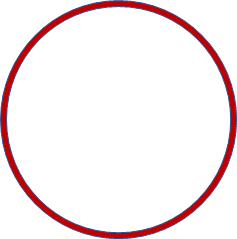

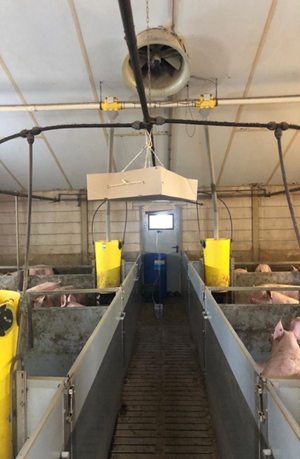
 At each farm we sampled the following compartments: a nursery compartment, a weaned piglets and a fattening pigs’/ older animal compartment, except for on two farms where only fattening pigs were present. At the farms where only fattening pigs were sampled, there was an effort made to place the EDCs and GSP samplers in the compartments housing the oldest and youngest animals available to get a range of information.


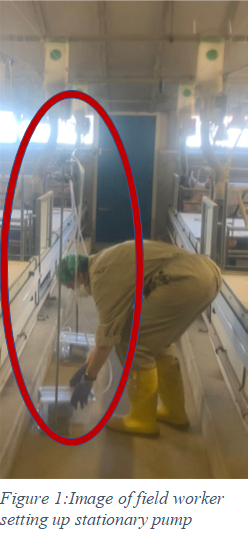
The EDCs were hung at a height of 1.5m to catch airborne matter and be out of reach of the animals (Figure S2). EDCs were then left in place for 7 days before being sent back by the farmer by mail to Utrecht University (the Netherlands) for laboratory analysis. This sampling duration was chosen as the large amount of dust found in farm settings can lead to overloading of the EDCs, and has been successfully employed in past studies (Madsen et al, 2019; Feld et al.,2017; Luiken et al., 2022). An average of two EDCs were hung in each stable acting as duplicate measurements. Additionally, a blank EDC was taken to each farm but remained sealed and was sent back and tested with the other samples and treated as a negative control. Once the EDCs arrived at the lab, both EDC cloths were sterilely removed from the plastic frame and placed in plastic zip top bags and stored at -20^o^C until analysis.

Figure S2: EDC hanging in a cardboard platform in a pig stable

### Active air sampling

Active air sampling was performed using Gilian Gilair 5 air sampling pumps along with Teflon filters (PE Drain Disc, Whatman: GE Healthcare, with Teflon 2.0µm 37mm filters: Pall Corporation) in GSP sampling heads. Pumps were calibrated before use and the flow (3.5 liters per minute) was checked after sampling to ensure consistency and to detect any malfunctioning pumps. At each farm a blank filter was transported to the farm but remained sealed and was used as a negative control. An average of two active samplers was placed in each stable. The GSP sampling heads were clamped onto a metal rod which was 1.5m tall (Figure S3). Any peculiarities, such as malfunctioning pumps or torn filters were recorded on the field forms.

Figure S3: A field worker setting up a GSP sampler

GSP samplers were left in place for 6 hours and checked periodically. When sampling was concluded, the GSP sampling heads were transported back to the lab and the filters were removed with sterilized tweezers and stored at -20C until analysis.

## Laboratory Methods

For analysis, EDCs were removed from -20^o^C storage, allowed to come to room temperature before both sides were placed into stomacher bags containing 20 ml FE buffer and blended with a stomacher for 10 minutes to suspend the material. The liquid was then transferred into a 50ml Falcon tube and centrifuged at 50g for 1 minute, the top 2ml of the resulting suspension was either used immediately or frozen at -20^o^C until DNA extraction could proceed. In total, 300 µl microliters of the EDC liquid was added to a tube containing 300 µl lysis buffer from the LGC mag kit (LGC genomics, Berlin, Germany) kit along with zirconia beads. Air filters were thawed and placed into tubes containing zirconia beads and 600µl lysis buffer was added. A buffer blank containing only 600µl lysis buffer was used as a control in each run. 500µl phenol was added to both tubes containing the air filters and beads, as well as to the tubes containing the EDC suspension and beads, and a control, all tubes were bead beaten for 4 minutes. The tubes were then spun for 20 minutes at 3200rpm at 7°C. 10 µl of magnetic beads were added to a deep well plate and the supernatant from the mixture spun in the previous step was added and mixed with the magnetic beads. This plate was then incubated for 60 minutes while being incubated at 24°C and shaken at 800rpm. After incubation the mixture was pipetted to mix and spun for 2 minutes at 500rpm. Next the deep well plate was held against a magnetic plate to isolate the magnetic beads and as much liquid as possible was removed before adding 200µl of Wash Buffer 1 supplied by the kit, to each well. The plate was then incubated again for 24 minutes at 800rpm, before being placed back on the magnet, Wash Buffer 1 is removed and 200 µl of Wash Buffer 2 from the kit was added to each well. The incubator was then set to 55°C and 800 rpm, where the plate was left for 40 minutes or until the pellet was dry. Once the plate appeared completely dry it was placed back on the magnet and 67 µl elution buffer was added to each well mixed with the magnetic bead pellet, before a last incubation at 55°C for 20 minutes unagitated. Finally, the plate was placed back on the magnet and taking care not to transfer any beads, the DNA isolate was transferred to a DNA storage plate, sealed, and stored at -20°C until it was used as template for the PCR. Five microliters of DNA isolate was used in the following quantitative Real Time PCRs targeting, *femA, nuc*, *mecA*, and 16S rRNA, with all samples being run in duplicate. All targets were detected using the LightCyler480 (Roche Molecular Biochemicals, Mannheim, Germany) and associated program. Each *femA/nuc/MecA* reaction consisted of 4 µl PCR grade water, 1 µl primer/probe mix and 10 µl LC480 probe mix (LightCycler^®^ 480 Probes Master Mix, Roche) and ran for 45 cycles. Each 16S rRNA reaction contained 7 µl PCR grade water, 1 µl primer 355F at 100uM, 1 µl primer 556R 10uM and 10 µl SYBER green master mix (iTaq™ Universal SYBR® Green One-Step Kit, Bio-Rad) and ran for 40 cycles. A negative (no template) and a positive control were included in each run.

Analysis of PCR Results

A reference strain of ST398 was cultured and the viable cells were enumerated and used in a DNA extraction. This DNA was serially diluted to make the calibration curves for each gene to express the PCR output (Ct values) as equivalent colony counts (CFUeq).

Table 1S: Formulas for CFUeq derived from the calibration curves for each gene target

| **PCR Gene Target** | **Formula** |
| --- | --- |
| 16S | CFUeq= 10^((mean Ct 16S – 40.22)/-4.043) |
| nuc | CFUeq= 10^((mean Ct nuc – 43.28)/-3.71) |
| femA | CFUeq= 10^((mean Ct femA – 41.89 )/- 3.547) |
| mecA | CFUeq= 10^((mean Ct mecA – 39.71 )/- 3.28) |
