# Supplementary material for: A comparison of passive and active dust sampling methods for measuring airborne methicillin-resistant Staphylococcus aureus in pig farms
Source: Ann Work Expo Health. 2023 Jun 10;67(8):1004–10. doi: 10.1093/annweh/wxad033 (PMC10516621; doi:10.1093/annweh/wxad033)
Supplement: Supplementary file 3 [file wxad033_suppl_supplementary_questionnaire_responses.docx]

A Comparison of Passive and Active Dust Sampling Methods for Measuring Airborne MRSA in Pig Farms: Questionnaire Responses Supplement

Authors: Anne Rittscher, Abel A. Vlasblom, Birgitta Duim, Peter Scherpenisse, Isabella van Schothorst, Inge M. Wouters, Liese Van Gompel, Lidwien A.M. Smit

Not part of the questionnaire (noted during field work)	Types of pigs present on the farm at time of sampling?	Piglets and sows, weaned piglets, fattening pigs	Piglets and sows, weaned piglets, fattening pigs	Piglets and sows, weaned piglets, fattening pigs	Fattening pigs only	Piglets and sows, weaned piglets, fattening pigs	Piglets and sows, weaned piglets	Fattening pigs only
1	Data sharing with ZLTO	yes	yes	yes	yes	n/a	left blank	n/a
2	How many people work there?	3	2	2	1	5	1 Full time 3 Part time	1
3	Average number of pigs on the farm?	950	1200 + 2400 piglets	left blank	1600	660 sows, 3600 piglets, 130 breeding males	left blank	3800
4	Number of sows per year?	127	600	440	n/a	660	310	n/a
5	Number of fattners per year?	40	600	3300	4960	left blank	no fatteners, 70 sows	11400
6	Type of production system?	1 week system	2 week system	1 week system	only fattening pigs	1 week system	1 week system	n/a
7	Any other livestock present on the farm?	yes	yes	yes	yes	no	yes	no
7a	a)if so what animals?	70 dairy cows and young, 200 ewes	3 roosters, 3 hens, pony, dog, cat	40 sheep	sheep	n/a	15 goats	n/a
8	Average age of weaning (days)?	27	24	24	27	27	26	28
9	Average age moved to fattening (days)?	65	63	70	73	73	3 months	73
10a	a)All in/all out used in the nursery unit?	yes	yes	yes	n/a	no	yes	n/a
10b	b)All in/all out used in the weaned unit?	yes	yes	yes	n/a	yes	n/a	n/a
10c	c)All in/all out used fattening unit?	no	yes	yes	yes	n/a	n/a	yes
11a	a)Type of feed given in the nursery unit?	dry	dry	dry	n/a	dry	dry	n/a
11b	b)Type of feed given in the weaned unit?	dry	dry	dry	n/a	dry	dry	n/a
11c	c)Type of feed given in the fattening unit?	dry	dry	dry	dry	n/a	n/a	wet and dry
12	Known probiotics in feed?	no	no	no	no	no	no	no
13	Name of food with probiotics?	n/a	n/a	n/a	n/a	n/a	n/a	n/a
14	Are the farrowing units cleaned or dininfected after each round?	cleaned & disinfected	cleaned	cleaned and disinfected	n/a	cleaned and disinfected	cleaned	n/a
15	Are the weaned units cleaned or dininfected after each round?	cleaned & disinfected	cleaned	cleaned and disinfected	n/a	cleaned and disinfected	no, not cleaned or disinfected	n/a
16	Are the fattening units cleaned or dininfected after each round?	cleaned & disinfected	cleaned	no (cleaned and disinfected only once per year)	cleaned and disinfected	n/a	no, not disinfected or cleaned	cleaned and disinfected
17a	Parts of the barn that are cleaned a)floors	yes	yes	yes	yes	yes	yes	yes
17b	b)windowsills cleaned?	yes	yes	yes	yes	yes	yes	yes
17c	c)feed/water pipes cleaned?	yes	yes	yes	yes	yes	left blank	yes
17d	d)walls cleaned?	yes	yes	yes	yes	yes	yes	yes
17e	e)other areas cleaned?	manure pit, pens	n/a	n/a	n/a	n/a	n/a	n/a
18	Which products are used to clean and disinfect ?	soaking agents and disinfectants	only water	soaking agents and disinfectants	soaking agents and disinfectants	soaking agents and disinfectants	soaking agents not disinfectants	soaking agents and disinfectants
19	Are corridors cleaned after pigs moved?	yes	yes	no	yes	yes	sometimes	yes
20	Is there a period of vaccancy after each round?	yes	weaned (3 days), fattening(7 days), nursery (never)	no	yes	yes	no	yes
21	Do workers always work from younger pigs to older?	yes	yes	yes	yes	yes	no	yes
22	Are nessaasry matierals along working lines?	yes	yes	no	yes	yes	no	yes
23a	Type of bedding used in the a) nursery unit?	sawdust, straw, ccm(corn cob mixture),	sawdust	sawdust	n/a	sawdust	none	n/a
23b	b) Type of bedding used with the weaned pigs?	none	alfalfa	none	n/a	sawdust	none	n/a
23c	c) Type of bedding used with the fattening pigs?	chopped straw	alfalfa	none	sawdust	n/a	none	flaxstraw
23d	d) How often is bedding changed in the nursery unit?	ccm daily, 7 days after birth	start of piglets	daily	n/a	Once	n/a	n/a
23e	e) How often is bedding changed for the weaned pigs?	n/a	daily	n/a	n/a	Once	n/a	n/a
23f	f) How often is bedding changed for the fattening pigs?	daily	daily	n/a	once when animals placed	n/a	n/a	once when animals placed
24	Type of ventillation used in the barn?	door ventilation	nursery(ground ventilation), weaned and fattening pigs (celing ventalation)	nursery(valve ventilation), piglets(ceiling ventilation), fattening(Well ventilation)	Other namely: Channel ventilation and feeding alley ventilation	Ceiling ventilation and other namly: Channel ventilation	Door ventilation	Other namely: Channel Ventilation
25	Maximum capacity of the weaned piglets?	3 or less per m2	3 or less per m2	wrote "according to guideline"	n/a	3 or less per m2	3 or less per m2	n/a
26	Maximum capacity of the fattening pigs?	1 or less per m2	1 or less per m2	b/w 0.7 and 0.9 m2 per pig	1 or less per m2	n/a	(sows) 1 or more m2 per sow	b/w 0.7 and 0.9 m2 per pig
